# Profiles of Peripheral Neuropathy and Risk Factors in People With Treatment-Naive Type 2 Diabetes Mellitus and Prediabetes

**DOI:** 10.7759/cureus.73304

**Published:** 2024-11-08

**Authors:** Mir Khalid Mairaj, Nazir Ahmad Pala, Mohd Ismail

**Affiliations:** 1 Department of Internal Medicine, Government Medical College Srinagar, Srinagar, IND

**Keywords:** diabetes and metabolic syndrome, diabetic peripheral neuropathy (dpn), new-onset diabetes, new-onset dia-betes, prediabetes, types 2 diabetes

## Abstract

Background: Diabetic peripheral neuropathy (DPN) is the most common complication of both type 2 diabetes mellitus (T2DM) and type 1 diabetes mellitus (T1DM) occurring in a majority of the affected people. DPN is the key initiating factor for the development of diabetic foot ulceration and the most common cause of non-traumatic lower-limb amputations. Whether chronic hyperglycaemia per se or altered levels of intermediate conventional risk factors in these people are responsible for the increased risk of DPN, is a matter of debate. There is little data about the profile of DPN in people with treatment-naive diabetes and prediabetes in the Asian population.

Objectives: This study aimed to determine the frequency of early DPN among people with newly detected treatment-naive diabetes and prediabetes attending a tertiary care public hospital. The study further aimed to determine the risk factors associated with DPN and to investigate whether such risk factors in these people might help to explain their increased risk of peripheral neuropathy.

Methods: A total of 65 consecutive people with treatment-naive T2DM with an equal number of people with prediabetes attending a tertiary care centre, diagnosed as per standard criteria, were enrolled in a non-randomised prospective study. The presence of peripheral neuropathy was assessed by the Michigan Neuropathy Screening Instrument (MNSI). Risk factor analysis for peripheral neuropathy in both groups was carried out. Statistical significance was considered with p-values of < 0.05.

Result: A total of 13 (20%) people with newly diagnosed treatment-naive T2DM and eight (12.3%) with prediabetes had DPN (MNSI score ≥ 2.5). Age (p < 0.001), high plasma glucose/glycated haemoglobin (HbA1c) (p < 0.001), BMI (p < 0.001), total cholesterol (p <0.001), albuminuria and hypertension (p < 0.001) were found to be strongly associated with early peripheral neuropathy.

Conclusion: DPN is a frequent complication among newly diagnosed treatment-naive people with T2DM and to a lesser extent among people with prediabetes. Various anthropometric, clinical, biochemical and metabolic factors in people with hyperglycaemia could account for the increased frequency of DPN in these people.

## Introduction

Diabetes is a group of metabolic diseases characterised by hyperglycaemia resulting from defects in insulin secretion, insulin action, or both [1]. Diabetic peripheral neuropathy (DPN) is the most common complication of both type 1 diabetes mellitus (T1DM) and type 1 diabetes mellitus (T2DM) and occurs in more than half of the affected individuals [2,3]. It is predominantly sensory neuropathy with autonomic nervous system involvement, although there are often motor features with advancing disease. DPN is the key initiating factor for the development of diabetic foot ulceration [4] and the most common cause of non-traumatic lower-limb amputations in most high-income countries [5]. Diabetic foot ulcers have been observed in nearly 25% of the patients during their course of illness [6]. The reported prevalence of diabetic foot ulceration in India is 12% [7]. Peripheral neuropathy, vasculopathy, and immunopathy are the main underlying mechanisms for diabetic foot complications, with peripheral neuropathy considered the main cause (>60%) of diabetic foot ulcers [8]. According to the WHO, lower-limb amputations are 10 times more common in people with diabetes than in people without diabetes [9]. 

Amputation is not only devastating in its impact on the individual and their family but also leads to loss of independence and livelihood. In low-income countries, the financial costs can be equivalent to 5.7 years of annual income, potentially resulting in financial ruin for individuals and their families [10]. Population-based studies (Western) showed that a substantial proportion of T2DM patients, often elderly patients who do not attend hospitals, suffered from peripheral neuropathy and peripheral vascular disease. These patients are at risk of foot ulceration and may benefit from preventive foot care [11]. These findings call for early detection of peripheral neuropathy, patient education, prompt management of minor foot injuries, provision of appropriate footwear, and community-directed programmes for earliest detection and intervention. The present study aimed to determine the frequency of early peripheral neuropathy among people with newly detected treatment-naive T2DM and prediabetes attending a tertiary care public hospital. The study further aimed to determine the risk factors associated with peripheral neuropathy and to investigate whether raised levels of such risk factors in these people might help explain their increased risk of peripheral neuropathy.

## Materials and methods

This study was conducted in the Department of Internal Medicine at Shri Maharaja Hari Singh (SMHS) Hospital, Government Medical College (GMC) Srinagar, Srinagar, India, over a period of two years. Sixty-five consecutive patients with newly detected treatment-naive T2DM with an equal number of people with prediabetes (total = 130) were included in the study. The study was approved by the Institutional Ethical Committee Government Medical College (GMC) Srinagar, and informed consent was obtained from all the study participants. Patients with newly diagnosed treatment-naive T2DM and prediabetes with ages ≥ 20 years to ≤ 60 years were enrolled. T2DM was diagnosed in accordance with the American Diabetes Association (ADA) criteria, where any of the following criteria were met: fasting plasma glucose (FPG) ≥ 126 mg/dl or two-hour plasma glucose (2-h PG) ≥ 200 mg/dl during an oral glucose tolerance test (OGTT) or glycated haemoglobin (HbA1c) ≥ 6.5% or a patient with classic symptoms of hyperglycaemia, or hyperglycaemic crisis, or random plasma glucose ≥ 200 mg/dl. Prediabetes was diagnosed by (HbA1c ≥ 5.7% and < 6.5% or FPG of 100-125mg/dl or impaired glucose tolerance (IGT): blood sugar results at the two-hour mark ≥ 140 and ≤ 199) [1].

People with T1DM, people with serious diabetic complications like lower limb amputation, patients with any symptoms suggesting nephropathy, and known smokers were excluded from the study.

Study protocol

Demographic details of patients like age, gender, duration of diabetes, previous history of foot ulcer, and history of any comorbidity were recorded. Blood pressure was calculated as the mean of two measurements performed in a sitting position after five minutes of rest, using a random-zero sphygmomanometer. Individuals were considered hypertensive if they had a diastolic blood pressure ≥ 90 mmHg, had a systolic blood pressure ≥ 140 mmHg, and/or were taking antihypertensive medication [12]. BMI was classified by the WHO criteria for the Asian population [13]. Diabetic neuropathy was measured by the Michigan Neuropathy Screening Instrument (MNSI), a reliable and validated tool for this purpose, where a total score of 0-10 was obtained. Vibration sensations were determined on the dorsum of the big toe by a turning fork (128 Hz). Each participant's feet were assessed by MNSI scoring for five defined parameters, which include appearance, ulceration, vibration sensations, ankle jerk, and touch pressure modality of sensation. Peripheral neuropathy was diagnosed when a patient on a 10-point scale had an MNSI score of ≥ 2.5 [14].

Metabolic parameters

An OGTT was performed with 75 g glucose dissolved in 250 ml water after an overnight eight-hour fast. Plasma glucose was estimated by an enzymatic method using glucose oxidase and peroxidase on an automated chemistry analyzer (Hitachi 912, Hitachi, Ltd., Chiyoda City, Tokyo, Japan). HbA1C was determined by high-performance liquid chromatography (HPLC). Lipid parameters were analysed with commercially available enzymatic reagents (Audit Diagnostics, Cork, Ireland) adapted to the Hitachi 912 autoanalyzer. The Friedewald formula was used to calculate low-density lipoprotein (LDL) cholesterol. The upper normal limit for the reference population for fasting total cholesterol, LDL, high-density lipoprotein (HDL), and triglycerides (TG) were 200 mg/dl, 100 mg/dl, 40 mg/dl, and 150 mg/dl, respectively. Serum creatinine was measured by the kinetic alkaline picrate method, blood urea nitrogen by the urease method, and urinary albumin was determined by a radioimmunoassay technique (Immunotech, Prague, Czech Republic).

Statistical analysis

The statistical software IBM SPSS Statistics for Windows, Version 20, (Released 2011; IBM Corp., Armonk, New York, United States) was used to analyse data. Continuous variables were expressed as mean ± SD and categorical variables as percentages. Student’s independent t-test was employed for comparing continuous variables. Chi-square or Fisher’s exact test, whichever was appropriate, was used for the comparison of categorical variables. All results have been described on a 5% level of significance, i.e., a p-value of < 0.05 is considered significant.

## Results

Among 130 patients (65 newly diagnosed treatment-naive T2DM with an equal number of people with prediabetes) recruited, peripheral neuropathy was detected in 13 (20%) people with newly diagnosed treatment-naive T2DM and eight (12.3%) in people with prediabetes. Mean (SD) age and BMI of people with T2DM with DPN were significantly higher (55.6 ± 4.5 vs 42.3 ± 7.5 years, p < 0.001 and 28.2 ± 3.0 vs 23.2 ± 3.2 kg/m2, p < 0.001, respectively) compared to those without DPN. Mean (SD) total cholesterol (343.9 ± 79.6 vs 216.5 ± 55.2 mg/dl, p < 0.001), FPG (246.3 ± 56.1 vs 182.2 ± 38.7 mg/dl, p < 0.001), postprandial plasma glucose (PPG) (367.2 ± 81.3 vs 292.6 ± 57.0mg/dl, p < 0.002), and HbA1c (10.1 ± 0.6 vs 7.8 ± 1.1, p < 0.001) at presentation was significantly higher in people with DPN compared to those without DPN. The frequencies of hypertension (92.3% vs 30.8%, p < 0.001) and albuminuria (76.9% vs 9.6%, p < 0.001) were significantly higher in people with T2DM and DPN compared to those without DPN (Table 1).

**Table 1:** Clinical and laboratory parameters of people with treatment-naive type 2 diabetes mellitus with and without diabetic peripheral neuropathy (DPN) (n=65). ^a^Data expressed as mean (SD).^ b^Data expressed as n (%) FPG: Fasting plasma glucose; PPG: Postprandial plasma glucose

Parameter	DPN (n=13)	Without DPN (n=52)	p-value
Age (years)^ a^	55.6 (4.52)	42.3 (7.57)	< .001
Total cholesterol (mg/dl)^ a^	343.9 (79.67)	216.5 (55.21)	< .001
(HbA1c%)^ a^	10.1 (0.695)	7.8 (1.184)	< .001
FPG (mg/dl)^ a^	246.3 (56.19)	182.27 (38.78)	< .001
PPG (mg/dl)^ a^	367.2 (81.37)	292.6 (57.02)	< .002
BMI (kg/m^2^)^ a^	28.2 (3.09)	23.2 (3.29)	< .001
Hypertension (mmHg)^ b^	12 (92.3)	18 (30.8)	< .001
Albuminuria^ b^	10 (76.9)	6 (9.6)	< .001

Similarly, people with prediabetes and DPN had significantly higher age and BMI (55.6 ± 4.5 vs 45.9 ± 8.4 years, p < 0.001 and 25.6 ± 2.0 vs 23.1 ± 2.8 kg/m2, p < 0.001, respectively. Mean (SD) total cholesterol (286.6 ± 58.0 vs 186.9 ± 37.7 mg/dl, p < 0.001), FPG (120.8 ± 1.9 vs 114.1 ± 6.4 mg/dl, p < 0.005), PPG (189.5 ± 4.3 vs 166.9 ± 16.4 mg/dl, p < 0.001), and HbA1C (6.2 ± 0.1% vs 5.9 ± 0.1%, p < 0.001) were significantly higher in people with DPN compared to those without DPN. The frequency of hypertension (100% vs 28%, p < 0.001) and albuminuria (100% vs 3.5%, p < 0.001) were significantly higher in people with DPN compared to those without DPN (Table 2).

**Table 2:** Clinical and laboratory parameters of people with prediabetes with and without diabetic peripheral neuropathy (DPN) (n=65). ^a^Data expressed as mean (SD). ^b^Data expressed as n (%) FPG: Fasting plasma glucose; PPG: Postprandial plasma glucose

Parameter	DPN (n=8)	Without DPN (n=57)	p-value
Age (years)^ a^	55.6 (4.52)	45.9 (8.47)	< .001
Total cholesterol (mg/dl)^ a^	268.6 (58.05)	186.9 (37.71)	< .001
(HbA1c%) ^a^	6.28 (0.138)	5.95 (0.198)	< .001
FPG (mg/dl)^ a^	120.8 (1.91)	114.1 (6.46)	< .005
PPG (mg/dl)^ a^	189.5(4.34)	166.9 (16.49)	< .001
BMI (Kg/m^2^)^ a^	25.6 (2.03)	23.1 (2.81)	< .001
Hypertension (mmHg)^ b^	8 (100)	18 (28)	< .001
Albuminuria^ b^	8 (100)	2 (3.5)	< .001

## Discussion

Frequency of DPN in people with newly diagnosed treatment-naive T2DM and prediabetes

Neuropathies may manifest early, during the first 12 months after the diagnosis of diabetes [15,16] even before any retinopathy can be detected by funduscopy [17]. Indeed, in the most recent statement of the ADA, it is reported that polyneuropathy should be expected in about one in 10 patients with T2DM at diagnosis [18]. Importantly, within the first year of diagnosis, its prevalence is expected to rise to 15.4% in T1DM and 43.4% in T2DM [15]. Neuropathies may be detected even earlier in patients with prediabetes. Indeed, polyneuropathy has been reported in 11% to 25% of patients with prediabetes [19,20]. The ADA has very recently accepted the presence of neuropathy in the setting of prediabetes mainly (impaired glucose tolerance) and has suggested polyneuropathy screening in those patients with prediabetes who have symptoms [2]. In our study, the prevalence of early peripheral neuropathy was 20% and 12.3%, respectively, in people with treatment-naive T2DM and prediabetes, which is consistent with the results from previous studies [15,19,20].

Risk factor analysis of peripheral neuropathy in people with prediabetes/diabetes

The current study was designed to recruit people with newly detected treatment-naive T2DM and prediabetes to detect peripheral neuropathy in the incipient stage and to analyse the risk factors associated with peripheral neuropathy in these people. With ageing, the resistance of the human body will decrease, the level of organ function will decrease, and the incidence of many diseases will increase. A cross-sectional study identified an older age as a risk factor for DPN [21]. The Diabetes Control and Complications Trial/Epidemiology of Diabetes Interventions and Complications (DCCT/EDIC) study also identified age as the most significant risk factor for DPN other than HbA1c levels [22]. Similar population-based studies in prediabetic subjects have identified older age as a significant risk factor for early peripheral neuropathy [19], which is consistent with the findings of the current study. The Diabetic Control and Complication Trial (DCCT) study [22] provided definite proof that reduction in chronic hyperglycaemia can prevent many of the early complications in patients with IDDM.

The United Kingdom Prospective Diabetes Study (UKPDS) [18]studied the course of about 5000 individuals with T2DM for more than 10 years. The UKPDS demonstrated that there was a continuous relationship between glycaemic control and the development of complications. Similarly, studies of prediabetic subjects [23,24] have demonstrated poor glycaemic control as a risk factor for early peripheral neuropathy, similar to the results of our study. Dyslipidemia is characterised by an abnormal plasma lipid profile with uncontrolled lipid levels, and both clinical and preclinical studies implicate a role for dietary fatty acids in the pathogenesis of neuropathy in diabetes and prediabetes. Molecular studies further show that saturated and unsaturated fatty acids differentially regulate the nerve lipid profile and nerve function [25]. Total cholesterol (TC) is the sum of the cholesterol contained in all lipoproteins in the blood. The analysis performed in this study concluded that TC was associated with DPN.

A study of 200 patients with T2DM showed a significant correlation between a TC > 5.2 mmol/L and DPN [26], similar to our study results. The plantar pressure is abnormal in T2DM patients presenting with DPN [27]. Some studies conducted in Western countries have shown that obesity (BMI > 30 kg/m2) causes plantar hypertension [28]. Therefore, obese patients with T2DM are more likely to suffer from DPN [18], which is consistent with our study results of high BMI associated with increased risk of neuropathy. Systolic blood pressure (SBP) is one of the risk factors for DPN among patients with T2DM. A systematic review showed a 2.6-fold higher SBP in T2DM patients with DPN than in T2DM patients without DPN [29]. Regardless of whether T2DM is accompanied by hypertension, an elevated SBP always increases the risk of DPN [30]. According to a study by Yokoyama et al. [31], the occurrence of diabetic neuropathy is significantly correlated with SBP. A study by Viswanathan et al. [24] of IGT subjects identified high SBP as the risk factor for early neuropathy. In our study, both elevated SBP and diastolic blood pressure (DBP) were associated with early peripheral neuropathy. In our study, albuminuria was found to be associated with peripheral neuropathy, similar to the results from previous population-based studies [32,33].

Limitations

The most important limitation of this study is the small sample size. Studies with larger sample sizes of people with newly diagnosed diabetes and more importantly of people with prediabetes are required to look for early peripheral neuropathy. Other causes of neuropathy like hypothyroidism and vitamin B12 deficiency have not been excluded from the study population.

## Conclusions

Peripheral neuropathy is a frequent complication among newly diagnosed treatment-naive people with T2DM and prediabetes. Various anthropometric, clinical, and biochemical parameters in people with hyperglycaemia could account for the increased frequency of DPN in these people. Early detection of DPN in people with newly diagnosed T2DM and prediabetes could help to devise a comprehensive foot care programme to minimize the risk of morbidity and mortality associated with DPN. Further studies with larger cohorts of people with newly diagnosed treatment-naive T2DM and prediabetes should be undertaken to confirm our observations.
